# Diverse model systems reveal common principles of meiosis

**DOI:** 10.1186/s13059-018-1527-9

**Published:** 2018-09-14

**Authors:** Weronika E. Borek, Adele L. Marston

**Affiliations:** Wellcome Centre Cell Biology, Institute of Cell Biology, School of Biological Sciences, University of Edinburgh, Edinburgh, EH9 3BF UK

## Abstract

A meeting report on the 14th Gordon Research Conference on Meiosis, held at Colby Sawyer College, New London, NH, USA, 9–15 June 2018, chaired by Monica Colaiacovo, Harvard Medical School.

## Introduction

Meiosis is a special type of cell division that generates gametes. The production of viable gametes relies on complex maneuverings of the genome that happen in the context of cellular differentiation events that vary between organism and sex. The meeting, entitled ‘Molecular mechanisms and regulation of meiosis across species’, discussed new advances stemming from studies on a range of model organisms. Here, we highlight the major themes that emerged. We apologize to the presenters of the many excellent talks that are not featured due to space restrictions.

## Initiation and regulation of the meiotic differentiation program

Exciting advances in understanding the signals that induce mouse male meiosis were presented. David Page (Whitehead Institute for Biomedical Research, Cambridge, MA) revealed the global role of the STRA8 protein, long known to be stimulated at meiotic entry, in upregulating a meiotic transcriptional program consisting of meiosis-specific and cell-cycle genes, whereas Kei-ichiro Ishiguro (Institute of Molecular Embryology and Genetics, Kumamoto, Japan) identified a potential upstream regulator of STRA8, called MEIOSIN. Satoshi Namekawa (University of Cincinnati College of Medicine, Cincinnati, OH) disclosed the dynamic landscape of super-enhancers during the mitosis-to-meiosis transition. Working in budding yeast, Gloria Brar (University of California, Berkeley, Berkeley, CA) provided insights into the surprising ‘sloppiness’ of the transcription and translation regulation throughout meiosis, in which cells ensure protein homeostasis of members of protein complexes by degradation, despite being capable of synthesizing components of protein complexes at stoichiometric levels.

## Insights into meiotic chromosome structure

A key feature of meiosis is the pairing and synapsis of homologous chromosomes. The synaptonemal complex (SC) is a conserved structure consisting of two lateral elements, a central element and transverse filaments that span the other elements to ‘zipper’ the homologs together. Although first visualized in 1956 by electron microscopy, the molecular details of its workings have only just begun to be uncovered. Kevin Corbett (Ludwig Institute for Cancer Research, San Diego, CA) described the structural conservation of lateral element proteins such as budding yeast Red1, human SYCP2-SYCP3, and *Arabidopsis* ASY3 and ASY4. In contrast to the long-held view of the SC as a static structure, Abby Dernburg (University of California, Berkeley, CA) demonstrated that different SC complex components show distinct diffusive behavior in *Caenorhabditis elegans*, leading to a model for the spatial patterning of crossovers (CO). Sean Burgess (University of California, Davis, CA) discussed the behavior of the SC in a novel meiotic model, *Danio rerio*, where its assembly begins at telomeres. Amy MacQueen (Wesleyan University, Middletown, CT) showed separable roles of the budding yeast SC protein Zip1 in chromosome synapsis and CO formation. The unique structure of meiotic chromosomes was also the theme of Matt Neale’s (University of Sussex, Brighton, UK) talk, which revealed the first genome-wide chromosome conformation capture (Hi-C) picture of early meiotic chromosomes from budding yeast. The appearance of multiple pachytene-specific interactions was indicative of the axis-loop structures formed at this stage, and dependent on axis proteins, including cohesin.

## Regulating double strand break formation

Meiotic recombination is initiated by the introduction of deliberate double strand breaks (DSBs) by the topoisomerase VI-related protein Spo11. DSBs are not randomly distributed, but are concentrated at preferred regions, called hotspots. The past few years have seen an advance in our understanding of the chromosomal features that typify hotspots. A collective highlight of the meeting was the progress made in understanding the connections between DSB formation, the chromosome axis, and synapsis. Scott Keeney (Memorial Sloan Kettering Cancer Center, New York, NY) and Attila Tóth (Technical University of Dresden, Dresden, Germany) both presented fascinating work on the mouse pseudautosomal region. This small region of homology between the X and Y chromosomes is necessary for their synapsis (and thus, meiosis) and undergoes high frequency DSB formation, but how this is assured was not known. These two labs identified a novel meiotic protein that is required for DSB formation and synapsis at this region. Jesus Page (Universidad Autónoma de Madrid, Madrid, Spain) focused on the evolution, and surprising divergence, of sex chromosomes in non-model mammals such as African pigmy mice.

In addition to locus-specific regulation, meiotic recombination is globally controlled. Maria Jasin (Memorial Sloan Kettering Cancer Center, New York, NY) described how the ATM checkpoint kinase regulates DSB numbers and proximity in mouse spermatocytes. Enrique (Fadri) Martinez-Perez (Imperial College, London, UK) postulated that in *C. elegans,* cohesins may regulate the kinase CHK-2 in pachytene through the SC. Needhi Bhalla (University of California, Santa Cruz, CA) explained how the *C. elegans* PCH-2 ATPase inhibits multiple meiotic processes through the effector HORMA domain proteins.

## Assuring crossovers

Meiotic recombination not only increases genetic diversity but also, critically, generates the crossover products that constitute linkages between homologous chromosomes. Ultimately, recombination must ensure sufficient and appropriately placed COs to join every homolog pair properly. Francesca Cole (University of Texas MD Anderson Cancer Center, Smithville, TX) used an elegant method to obtain highly synchronized spermatocytes and to map COs and non-crossovers and the time of their appearance, leading to key predictions about the mechanism of DSB repair and CO assurance. The role of the CO factor, SUMO/ubiquitin ligase, Zip3, in meiosis is only beginning to be understood. Scott Hawley (Stowers Institute for Medical Research, Kansas City, MO) presented beautiful work on the newly discovered *Drosophila* Zip3 homologs, Vilya, Narya, and Nenya, and their roles in DSB and CO formation. Gerry Smith (Fred Hutchinson Cancer Research Center, Seattle, WA) discussed the role of linear element proteins (that are related to the SC in other species) in limiting the number of DSBs by clustering hotspots in *Schizosaccharomyces pombe*, thus providing a molecular basis for CO interference. Denise Zickler (Institute of Integrative Cell Biology (I2BC), Gif-sur-Yvette, France) elucidated the roles of *Sordaria macrospora* Zip2 and Zip4 proteins in CO formation. Nancy Kleckner (Harvard University, Cambridge, MA), in collaboration with the Wang-Zhang laboratory (Shandong University, Jinan, China), observed that organisms vary their CO levels on a per-nucleus basis, and that they do so by varying their chromosome axis length. She suggested that this effect results from global modulation of chromatin loop sizes. This variation leads to a broad distribution of the numbers of COs per nucleus, and might be a mechanism to reconcile the opposing effects of recombination, which can both create evolutionarily favorable allele combinations and disrupt existing combinations that have been selected to be favorable over time. Indeed, as revealed by Neil Hunter (University of California, Davis, Davis, CA) talking about SUMO-modifications in meiosis, changes in axis length affect CO number in mice.

## New technologies advancing our understanding of meiosis

Recent technologies relying on high-throughput sequencing have revolutionized our understanding of recombination outcomes and their molecular mechanisms. Expanding on ‘calibrated chromatin immunoprecipitation (ChIP)’, developed in the Nasmyth lab (University of Oxford, Oxford, UK), Andreas Hochwagen (New York University, New York, NY) introduced SNP-ChIP, in which he used single nucleotide polymorphisms (SNPs) between diverged *Saccharomyces cerevisiae* strains to identify the spiked-in DNA used for calibration after sequencing. Franz Klein (University of Vienna, Vienna, Austria) showed a similar calibration method, but used SNPs between different species, *S. cerevisiae* and *S. kudriavzevii*. Both strategies now allow calibration between samples from ChIP of meiosis-specific, untagged proteins. Franz Klein also devised Protec-Seq to map double-stranded DNA fragments that are protected by Spo11, which result from the unexpected occurrence of coordinated pairs of DSBs. Dan Camerini-Otero (National Institute of Diabetes and Digestive and Kidney Diseases, Bethesda, MD) developed an elegant method to map replication origins in mouse testes and correlated the usage of DSB hotspots to early replicating domains. Michael Lichten (National Cancer Institute, Bethesda, MD) employed a synthetic recombination hot-spot with evenly and densely spaced SNPs together with high-throughput sequencing to map parental strand contributions to meiotic recombinants. The analysis, performed in a mismatch-repair mutant background to preserve mismatches, revealed the unexpected complexity of meiotic recombination outcomes. For example, template switching was found to be surprisingly common, whereas simple synthesis-dependent strand annealing was infrequent. In addition, novel methods that were not sequencing-based were also developed. Melina Schuh (Max Planck Institute for Biophysical Chemistry, Göttingen, Germany) described a newly developed method, TrimAway, that allows the rapid degradation of protein in oocytes in vivo. Barbara Meyer (University of California, Berkeley, Berkeley, CA), who was presented with the Thomas Hunt Morgan Medal by the Genetics Society of America for lifetime achievement in genetics by Anne Villeneuve (Stanford University School of Medicine, Stanford, CA) during the meeting, outlined strategies for using CRISPR/Cas9 in *C. elegans*.

## Unique features of the meiotic cell cycle and cytoskeleton

Historically, the meiosis field has focused largely on meiotic recombination, leaving vast areas of meiotic cell biology unexplored. This meeting highlighted the increased interest in some of the unusual aspects of meiosis. Roberto Pezza (University of Oklahoma Health Science Center, Oklahoma City, OK) discussed proteins that are required for the telomere-led rapid prophase movements (RPMs) of chromosomes, an essential process occurring in meiotic prophase that helps to establish homologue interaction during pairing. Owen Davies (Newcastle University, Newcastle, UK) presented a crystal structure of the human MAJIN-TERB2 complex, elucidating the molecular basis of telomere–nuclear envelope interactions, which facilitate RPMs. Hiro Ohkura (University of Edinburgh, Edinburgh, UK) identified a new pathway for microtubule nucleation in meiosis I *Drosophila* oocytes requiring the kinesin Subito, which compensates for the absence of major microtubule organizers—centrosomes. Tomoya Kitajima (RIKEN Center for Biosystems Dynamics Research, Kobe, Japan) showed unexpected roles of kinetochore subcomplexes in the assembly of the meiosis I, but not the meiosis II, spindle in mouse oocytes. Sadie Wignall (Northwestern University, Evanston, IL) discussed the kinetochore-independent chromosome segregation of *C. elegans* oocytes and explained how SUMOylation promotes the assembly of the ring complex that compensates for kinetochores in this system. Raphael Mercier (Université Paris-Saclay, Versailles, France) identified a key cell cycle regulator in *Arabidopsis* that is important for chromosome segregation. Elçin Ünal (University of California, Berkeley, Berkeley, CA) presented unexpected findings on mitochondrial segregation and inheritance. Soni Lacefield (Indiana University, Bloomington, IN) entertained the audience with beautiful videos of budding yeast mutants that continue to divide after meiosis II, and was congratulated by a questioner on her discovery of ‘Meiosis III’.

## Conclusions and perspectives

A future challenge for the field is to apply our increasing molecular knowledge of meiosis in model systems to reveal the origin of aneuploidy in human gametes. Although the scarcity of research material has prohibited this in the past, partnerships between scientists and in vitro fertilization clinics are beginning to address this issue, as discussed by Eva Hoffmann (University of Copenhagen, Copenhagen, Denmark) at the meeting. These future challenges will be addressed during the next GRC Meiosis meeting in 2020, which will be chaired by Paula Cohen (Cornell University, Ithaca, NY) and co-chaired by Jeff Sekelsky (University of North Carolina, Chapel Hill, NC).

Overall, this meeting showcased how a remarkable diversity in research topics and model systems enrich the field of meiosis and provided the ideal setting for common principles to emerge.

## Abbreviations

ChIP: Chromatin immunoprecipitation
CO: Crossover
DSB: Double strand break
RPM: Rapid prophase movement
SC: Synaptonemal complex
SNP: Single nucleotide polymorphism

